# Can Humans Discriminate Horse ‘Fear’ Chemosignals from Control Chemosignals? Comment on Sabiniewicz et al. A Preliminary Investigation of Interspecific Chemosensory Communication of Emotions: Can Humans (*Homo sapiens*) Recognise Fear- and Non-Fear Body Odour from Horses (*Equus ferus caballus*). *Animals* 2021, *11*, 3499

**DOI:** 10.3390/ani12121489

**Published:** 2022-06-08

**Authors:** Gün R. Semin, Nuno Gomes, Biagio D’Aniello, Agnieszka Sabiniewicz

**Affiliations:** 1 William James Center for Research, ISPA–Instituto Universitário, 1149-041 Lisbon, Portugal; ngomes92@gmail.com; 2 Faculty of Social and Behavioral Sciences, Utrecht University, 3584 CS Utrecht, The Netherlands; 3 Department of Biology, University of Naples Federico II, Via Cinthia, 80126 Naples, Italy; biagio.daniello@unina.it; 4 Department of Otorhinolaryngology, Smell and Taste Clinic TU Dresden, 01307 Dresden, Germany; a.sabiniewicz@gmail.com

## Abstract

We illustrate the problematic nature of different assumptions guiding the examination of whether humans can detect the source of fear chemosignals (i.e., body odors) emitted by horses—a research question examined in an article recently published in *Animals*. A central issue is that the formulation of the question itself contains the answer to it. In this paper, we parse the problematic assumptions on which the analysis and methodology rely, leading to conclusions that are difficult to support. These assumptions constitute examples of methodological problems that should be avoided in research with animals and odors. The unique aspect of this paper is that it is a collaborative product, including the original contributor, in the pursuit of transparency in science.

## Can Humans Discriminate Horse ‘Fear’ Chemosignals from Control Chemosignals? A Methodological Comment

Questions about replicability and methodological rigor have been a general concern in psychological research for some time now. These concerns have spread out to various empirical disciplines, ranging from biology to medicine. Some of these problems have also been articulated for research on olfactory processes [1], referring, in this case, to potential false positives. Wyatt signals that similar issues exist in research on the effects of olfactory cues on human behavior. The goal of the current contribution is to export one of these emerging issues in a collaborative effort to bring scientific transparency to a research paper authored by one of the present authors (A. Sabiniewicz) and her collaborators. This unique contribution draws attention to the dangers arising from the formulation of dependent variables in animal research, potentially leading to false conclusions. We use the example to illustrate this point is Sabiniewicz et al. (2021) [2].

Sabiniewicz and colleagues (2021) recently published an exciting study arguing that humans can identify fear sweat emitted by horses. The argument rests on their report suggesting that humans could differentiate the chemosignals of horses experiencing fear from the chemosignals collected while they were not experiencing this emotion (control). We know of studies that have documented the communication of fear and happiness experiences via the body odors of human beings to pet dogs (*Canis lupus familiaris*) (e.g., D’Aniello et al., 2018; 2021) [3,4] and horses (*Equus ferus caballus*) (Semin et al., 2019; Sabiniewicz et al., 2020) [5,6]. Still, Sabiniewicz and her colleague’s Field (2021) study is the first to show the reverse, namely, from horses to humans. It, therefore, opens a potentially novel insight into the possible bidirectional nature of the chemosensory medium. For this reason alone, it is essential to carefully examine the reported research to know if it has potential limitations that question the validity of its findings.

From our perspective, three main issues may constitute potential confounds to the results reported by Sabiniewicz and colleagues (2021), making it difficult to conclude that humans knowingly discriminate between fear sweat emitted by horses and their ‘control sweat’. The first is whether the horses were experiencing fear. The sweat of the horses in the race condition (*“stress related to carrying a rider and taking part in a race”*; Sabiniewicz et al., 2021, p. 3), which is described as the ‘fear’ condition, is compared with sweat obtained from the same horses in a walking condition. The two were separated by at least 20 min. The presence or absence of fear is supposed to be indicated by reference to *“ear position, head position, body tension, and behavior of the horses”* (Sabiniewicz et al., 2021, p. 3). The authors do not supply any concrete evidence. It is unclear what the horses are experiencing, and the authors interchangeably use the terms “stress” and “fear”. Given the absence of empirically demonstrable operationalization, one can advance a simple and equally plausible alternative. The difference between the two conditions is simply in cardio activation. In short, the central pillar of the paper is arguably fragile. The authors themselves argue that: “*the horses’ reactions in the fear condition were only validated via behavioral measures. Thus, it may be insufficient to conclude whether this condition generated fear and, if so, to what extent*” (Sabiniewicz et al., 2021, p. 5). There is no objective report in the paper of what these behavioral measures yielded.

The second obstacle, and main problem, is the formulation of the central dependent variable. An essential requirement in formulating questions in research is not to provide the answer to a question by the formulation of the question (Semin & De Poot, 1997) [7]. In Sabiniewicz et al. (2021), the option for the human participants was to decide “*which of the two jars contained the fear odor and which one contained the non-fear odor*” (emphasis here; Sabiniewicz et al., 2021, p. 3). This question assumes that participants have identified the fear odor, which is highly unlikely, if not impossible. The participants have, likely, no clue that the odor is one of fear. The question informs the participants that one of the jars definitively contains a *fear* odor. The replies will always be interpreted as “fear”. If the very same sweat samples were given to participants and they were asked to judge which of the two jars contained anger or disgust, then the dominant responses would have been anger or disgust. In fact, the participants would not have been able to say that the samples were horse sweat. The solution to this problem is to find systematic nonbiased sequences of questions.

The obstacle presented by the inopportune question formulation is compounded by the second question regarding whether the “*odor pair were equally intense or if one was more intense than the other*” (Sabiniewicz et al., 2021, p. 3). When identifying the fear versus no fear odors, the average accuracy was 65.7%. Interestingly, the fear odor was judged as more intense 64% of the time. The odor intensity likely provided the cue driving the answers to the biased question regarding the presence or absence of fear. The intensity of the odor was guessed to indicate which of the two odors was from fear and which was not. In other words, having a binary option of identifying an odor as “fear or not” may have led participants to select the more intense odors as the ones containing “information” and, thus as the ones associated with fear, which was the only answering option they got. Hence, this may have produced the obtained results, even if the participants could not process emotional information in the horses’ chemosignals. With this in mind, we reanalyzed Sabiniewicz and colleagues’ data (2021) using jamovi (The jamovi project, 2019) [8] and the GAMLj module (Gallucci, 2019) [9]. First, we examined a possible correlation between the number of trials where the fear odor was identified “correctly” and the number of trials where the fear odor was rated as more intense than the control. If intensity drove participants’ answers in identifying the fear odor, these two variables should be positively associated. A Spearman’s rank-order correlation confirmed this association. As predicted, a positive, even if not strong, correlation was evidenced (*r_s_* = 0.23, *p_correlated positively_* = 0.023, *n* = 73), showing that the higher the number of trials where fear was rated as more intense, the higher the number of “correct” fear odor identifications. Next, we continued to explore the possible confounder introduced by odor intensity, examining the probability of correctly identifying fear odors in (1) trials where the fear odor was identified as more intense; (2) trials where the fear and control odors were rated as equally intense; (3) trials where the control odor was rated as more intense than fear. This was examined using a logistic mixed-effects model, which predicted the probability of the fear odor being correctly identified (0 = control identified as the fear odor; 1 = fear odor correctly identified) in terms of log odds. Intensity assessments [i.e., fear more intense (*N_trials_* = 752); control more intense (*N_trials_* = 181); both equally intense (*N_trials_* = 235)] were entered as a predictor to the model. The Participant ID and Odor ID (i.e., which samples were used in each trial) were included as random intercepts to control for possible interindividual and interstimulus variability. The model (*n* = 73; *R*^2^*_Marginal_* = 0.10; *R*^2^*_Conditional_* = 0.15) revealed that fear odor identification was shaped by the intensity assessments [*χ*^2^(2) = 83.78, *p* < 0.001]. As evidenced by post hoc tests, the probability of identifying the fear BO was significantly higher when the fear odor was rated as being the more intense (*Prob*. = 0.76; *SE* = 0.02) than when the control was the more intense (*Prob*. = 0.37; *SE* = 0.04; *z* = 8.92, *p_bonferroni_* < 0.001) or when they were rated as equally intense (*Prob*. = 0.60; *SE* = 0.04; *z* = 4.35, *p_bonferroni_* < 0.001). Interestingly, the probability of correctly identifying the fear odor when both odors were rated as equally intense was also significantly higher than when the control was the more intense one (*z* = 4.38, *p_bonferroni_* < 0.001). From our perspective, it is possible to conclude from these analyses that the effects evidenced by Sabiniewicz and colleagues (2021) were modulated, at least in part, by the reported intensity of the odor samples. Instead of a binary choice, a continuous measure of intensity would help quantify the extension of the confounder introduced here by this variable.

The methodological problems noted above highlight essential problems that must be faced in research on the bidirectionality of interspecies communication of emotions via chemosignals. Furthermore, they indicate that a conclusion such as “*humans, as a group, were able to correctly assign whether horse odor samples were collected under a fear- or a non-fear condition, respectively*” (Sabiniewicz et al., 2021, p. 5) is difficult to sustain without attending to the aforementioned issues. One possible and straightforward way would be to ask participants to smell the odors, including some dummy odors, one by one in a randomized order, and then to ask each participant to name what they think each odor is freely. This format of questioning would avoid any bias.

## Data Availability

Data available from the fourth author.
